# Dispersion of evaporating cough droplets in tropical outdoor environment

**DOI:** 10.1063/5.0026360

**Published:** 2020-11-01

**Authors:** Hongying Li, Fong Yew Leong, George Xu, Zhengwei Ge, Chang Wei Kang, Keng Hui Lim

**Affiliations:** A^*^STAR Institute of High Performance Computing, 1 Fusionopolis Way, Connexis 138632, Singapore

## Abstract

The ongoing Covid-19 pandemic has focused our attention on airborne droplet transmission. In this study, we simulate the dispersion of cough droplets in a tropical outdoor environment, accounting for the effects of non-volatile components on droplet evaporation. The effects of relative humidity, wind speed, and social distancing on evaporative droplet transport are investigated. Transmission risks are evaluated based on SARS-CoV-2 viral deposition on a person standing 1 m or 2 m away from the cougher. Our results show that the travel distance for a 100 *µ*m droplet can be up to 6.6 m under a wind speed of 2 m/s. This can be further increased under dry conditions. We found that the travel distance of a small droplet is relatively insensitive to relative humidity. For a millimetric droplet, the projected distance can be more than 1 m, even in still air. Significantly greater droplets and viral deposition are found on a body 1 m away from a cougher, compared to 2 m. Despite low inhalation exposure based on a single cough, infection risks may still manifest through successive coughs or higher viral loadings.

## I. INTRODUCTION

The severe acute respiratory syndrome coronavirus SARS-CoV-2 pandemic has spread worldwide progressively since it was first identified in Wuhan, China, in December 2019.^1^ Although the exact transmission mechanism of SARS-CoV-2 remains unclear,^2,3^ it is generally agreed that the airborne transmission route cannot be dismissed.^4^ Respiratory viruses including SARS-CoV-2 can be dispersed through droplets expelled from an infected person during coughing, sneezing, talking, and even breathing.^2^ Face touching is a potential secondary transmission mechanism of SARS-CoV-2,^5^ and direct inhalation of virus-laden droplets or droplet nuclei is another.^2,6,7^

Several studies have reported the size distributions of droplets generated through expiratory activities^8–12^ in terms of their diameters.^13^ Large droplets refer to those with a diameter larger than 100 *µ*m,^5^ and they tend to settle quickly due to gravity. In contrast, smaller droplets remain suspended for longer periods of time and may evaporate into aerosols or droplet nuclei, presenting long range transmission risk. The spreading of viruses through aerosols and droplet nuclei is referred to as “airborne transmission.”^14^

The dispersion range of cough droplets remains controversial. According to the seminal work of Wells,^13^ 100 *µ*m droplets settle within a horizontal distance of 2 m from a cougher. However, Xie and co-workers^15^ found that droplets could travel further than 6 m based on a characteristic jet velocity of 50 m/s from a sneeze; even with a slower cough velocity of 10 m/s, droplets can still travel substantially further than 2 m. Recent work by Bourouiba^3^ showed that expiratory activities, such as sneezes and coughs, release a turbulent cloud of buoyant gas with suspended droplets of various sizes. Such gas clouds can suspend airborne droplets up to distances of 7 m–8 m before losing momentum. Both droplet trajectories and evaporation rates are strongly affected by the gas cloud. Compared to large droplets, smaller droplets are suspended by the buoyant gas cloud and transported over long distances. These droplets may be vehicles for pathogens and therefore pose potential risks to susceptible hosts at a distance. A recent study reported that SARS-CoV-2 can remain viable in aerosol for up to 3 h during their experiment.^16^ Another study showed that SARS-CoV-2 positive particles with sizes smaller than 4 *µ*m are detected in hospital rooms of the infected patients.^17^ Therefore, understanding the airborne behavior for both large and small droplets is critical to reduce infection risks and break the transmission chain of SARS-CoV-2 infection.

Droplet trajectories are strongly affected by aerodynamics. To better understand SARS-CoV-2 transmission, it is critically important to fully understand the flow dynamics for both air flow and droplets including their interactions as well as droplet evaporation. For instance, under conditions of high temperature and low relative humidity (RH), a droplet could evaporate and shrink, which, in turn, affects its trajectory and eventual fate. In addition to experimental studies on aerosol dispersion, theoretical and numerical simulations play a useful complementary role. Specifically, numerical simulations provide an accurate prediction of droplet motion, and issues regarding the evaporation of droplet need to be addressed. Given the large number of droplets expelled from the expiratory activities, the Eulerian–Lagrangian approach is suitable and popular,^18^ although droplet–air interfaces are not computationally resolved due to computational costs.

Several Eulerian–Lagrangian numerical simulations have been conducted and reported since the outbreak of SARS-CoV-2.^19–25^ Blocken *et al.*^19^ investigated droplet trajectories for the runners’ geometries in the absence of external wind and showed that a significant number of droplets are present in the slipstream of the leading runner. Dbouk and Drikakis^20^ investigated airborne transmission in an open space environment and showed that the droplets can travel up to 6 m with the wind speed from 1.1 m/s to 4.2 m/s. Simulations of airborne transmission in a grocery store showed that the airborne cloud spread from the coughing person in the aisle to the immediate vicinity in a few minutes.^21^ Feng *et al.*^22^ investigated the dispersion of cough droplets under different wind velocities and relative humidity (RH) and showed that the droplet travel distance was highly dependent on the environmental conditions. Similar conclusions were reached by Pendar and Páscoa^23^ who performed numerical simulations for sneeze droplets’ transmission in an indoor environment. Wang *et al.*^24^ found that the droplet lifetimes and transport distances are significantly affected by the environment conditions. Taken together, these studies raise concerns over the effectiveness of social distancing, which ranges from 1 m^26^ to 2 m.^27^ Some simulation studies have suggested that a social distancing of 2 m may not be sufficient.^19–23^

In view of the recent easing of restrictions, such as phase 2 in Singapore,^28^ we believe that understanding airborne transmission of SARS-CoV-2 could help prevent the secondary or multiple infection waves. Currently, the effects of non-volatile components such as pathogens and salt on the droplet evaporation rate are not well understood.^29^ There is a recent modeling study on the effects of non-volatile components and reaction kinetics on the evaporation rate of the droplet,^25^ but the role of the droplet composition in evaporative dispersion mechanisms remains unclear.

In the present study, we model numerically the evaporative droplet dispersion under different tropical outdoor environmental conditions, including the effects of non-volatile components on the droplet flight simulations, which to our knowledge, has not been reported elsewhere. We believe our study is of value to both scientists and policy makers in our efforts to understand and mitigate the transmission of SARS-CoV-2 in our global community.

## II. METHODS

**FIG. 1.**
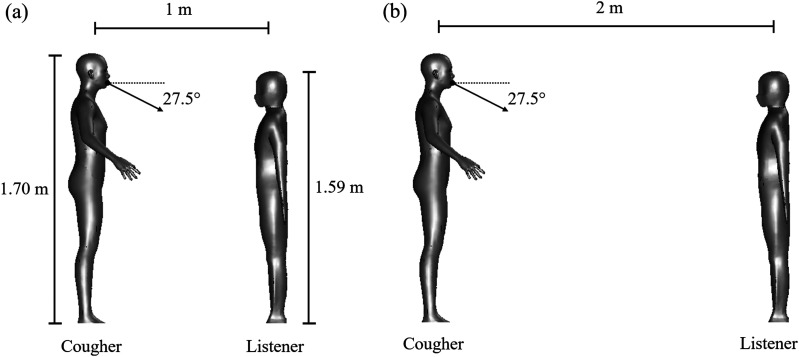
Schematic diagram for two standing persons (left: “cougher”; right: “listener”) spaced (a) 1 m and (b) 2 m apart. The heights of the cougher and listener are 1.70 m and 1.59 m, respectively. The arrow indicates the reference inclination of the cough jet from the horizontal.

We consider two standing persons in a tropical outdoor environment. One initiates a sudden cough and is labeled “cougher,” and the other is “listener.” The two persons maintain a social distance of 1 m and 2 m, as shown in Figs. 1(a) and 1(b), respectively. The height of the cougher and listener are 1.70 m and 1.59 m, respectively. The cougher coughs and expels a downward jet with a typical flow rate at an angle of 27.5°.^30^ After cough, the cougher begins normal breath. The listener remains normal breath in the whole process. The same breath cycle proposed by Bulińska and Buliński^31^ are used for the two persons. The breath temperature is 36 °C with saturated vapor, i.e., relative humidity RH = 100% at the mouth region. The droplets expelled by the cougher have a standard size distribution ranging from 2 *µ*m to 1000 *µ*m.^8^ Since the exact composition of mucosalivary fluid is unclear, salt is assumed to be the only non-volatile component in the droplet in this study.

### A. Theory

The problem involves fluid flow and heat transfer, species transport, and droplet movement as well as droplet evaporation. The governing equations are as follows:

#### 1. Fluid dynamics and heat transfer

The governing equations for fluid mass and momentum with turbulence are

$$\frac{\partial \rho }{\partial t}+\nabla \cdot \left(\rho \vec{u}\right)=\dot{m},$$ (1)

$$\frac{\partial \left(\rho \vec{u}\right)}{\partial t}+\nabla \cdot \left(\rho \vec{u}\vec{u}\right)=-\nabla P+\nabla \cdot \left[\left(\mu +\mu_{t}\right)\left(\nabla \vec{u}+\nabla {\vec{u}}^{T}\right)\right]-\;\nabla \cdot \left(\frac{2}{3}\rho \kappa \vec{I}\right)+F_{m},$$ (2)

where *κ* is the turbulent kinetic energy and *ε* is the dissipation of turbulent energy, expressed as

$$\;\frac{\partial \left(\rho \kappa \right)}{\partial t}+\nabla \cdot \left(\rho \vec{u}\kappa \right)=\nabla \cdot \left(\frac{\mu_{t}}{\sigma_{k}}\nabla \kappa \right)+G_{k}-\rho \varepsilon ,$$ (3)

$$\frac{\partial \left(\rho \varepsilon \right)}{\partial t}+\nabla \cdot \left(\rho \vec{u}\varepsilon \right)=\nabla \cdot \left(\frac{\mu_{t}}{\sigma_{\varepsilon }}\nabla \varepsilon \right)+\frac{\varepsilon }{\kappa }\left(C_{1\varepsilon }G_{k}-C_{2\varepsilon }\rho \varepsilon \right),$$ (4)

where *C*_1*ε*_, and *C*_2*ε*_ are constants 1.44 and 1.92, respectively, and *σ*_*κ*_ and *σ*_*ε*_ are 1.00 and 1.3, respectively.^32^ *G*_*k*_ is the production of turbulence kinetic energy.

Eddy viscosity *μ*_*τ*_ is expressed as

$$\mu_{t}=\rho C_{\mu }\frac{\kappa^{2}}{\varepsilon },$$ (5)

where *C*_*μ*_ is equal to 0.09.

The source terms in continuity and momentum equations (1) and (2) account for fluid loss via evaporation,

$$\;\dot{m}=\frac{\Delta m_{d}}{m_{d0}}\frac{{\dot{m}}_{d0}}{V},$$ (6)

$$F_{m}=\frac{1}{V}\left(\sum \left(\frac{18\mu C_{D}\text{R}{\text{e}}_{D}}{24\rho_{d}\overset{2}{\underset{d}{D}}}\left({\vec{u}}_{d}-\vec{u}\right)\right){\dot{m}}_{d}\Delta t\right),$$ (7)

where *m*_*d*0_ is the mass of the droplet, ${\dot{m}}_{d0}$ is the rate of change of droplet mass, and *V* is the control volume.

In addition to the flow field, species transport equations are also solved. Air is assumed to consist of three main species, i.e., O_2_, N_2_, and H_2_O vapor. The mass fractions of O_2_ and H_2_O are solved by

$$\frac{\partial \left(\rho x_{i}\right)}{\partial t}+\nabla \cdot \left(\rho \vec{u}x_{i}\right)=\nabla \cdot {\vec{J}}_{i}+S_{i},$$ (8)

where ${\vec{J}}_{i}$ is the diffusive flux of species *i* and can be expressed as

$${\vec{J}}_{i}=-\left(\rho D_{i,m}+\frac{\mu_{t}}{Sc_{t}}\right)\nabla x_{i}-D_{t}\frac{\nabla T}{T},$$ (9)

where *Sc*_*t*_ is the turbulent Schmidt number (taken as 0.7) and *D*_*t*_ is the turbulence diffusivity. The source term of species *i* is simply

$$S_{i}=\frac{dm_{d}}{dt}\frac{1}{V}.$$ (10)

The energy conservation equation is

$$\frac{\partial \left(\rho E\right)}{\partial t}+\nabla \cdot \left(\rho \vec{u}E\right)=\nabla \cdot \left(\left(\lambda \nabla T\right)-\underset{i}{\sum }h_{i}{\vec{J}}_{i}\right)+S_{h},$$ (11)

where *E* is the energy,

$$E=h-\frac{P}{\rho }+\frac{{\vec{u}}^{2}}{2},$$ (12)

*h* is the sensible heat,

$$h=\underset{i}{\sum }x_{i}C_{p,i}T+\frac{P}{\rho },$$ (13)

and *S*_*h*_ is thermal source term,

$$S_{h}=\frac{1}{V}\left(\frac{{\dot{m}}_{d0}}{m_{d0}}\left(m_{d,in}-m_{d,out}\right)h_{fg}+m_{d,in}C_{pd}T_{in}-m_{d,out}C_{pd}T_{out}\right),$$ (14)

where the subscripts *in* and *out* identify the droplets entering and exiting a control volume.

#### 2. Droplet tracking model

The equation of motion of a droplet (subscript *d*) is

$$\frac{d{\vec{u}}_{d}}{dt}=-F_{D}\left({\vec{u}}_{d}-\vec{u}\right)+\frac{\vec{g}\left(\rho_{d}-\rho \right)}{\rho_{d}},$$ (15)

where ${\vec{u}}_{d}$ and $\vec{u}$ are the droplet and air velocities, respectively. *F*_*D*_ is the drag force,

$$F_{D}=\frac{18\mu }{\rho_{d}D_{d}^{2}}\frac{C_{D}\text{Re}}{24},$$ (16)

where *D*_*d*_ is the droplet diameter and *C*_*D*_ is the drag coefficient as a function of the droplet Reynolds number,^33^

$$C_{D}=c_{1}+\frac{c_{2}}{\text{Re}}+\frac{c_{3}}{\text{R}{\text{e}}^{2}},$$ (17)

$$\text{Re}=\frac{\rho D_{d}\left|{\vec{u}}_{d}-\vec{u}\right|}{\mu },$$ (18)

where *c*_1_, *c*_2_, and *c*_3_ are empirical constants for spherical droplets estimates at the following Reynolds number intervals:

$$c_{1},c_{2},c_{3}=\left\{\begin{array}{cccc} 0, & 24, & 0, & 0<\text{Re}<0.1\\ 3.69, & 22.73, & 0.0903, & 0.1<\text{Re}<1\\ 1.222, & 1.667, & -3.889, & 1<\text{Re}<10\\ 0.6167, & 46.5, & -116.67, & 10<\text{Re}<100\\ 0.3644, & 98.33, & -2778, & 100<\text{Re}<1000\\ 0.357, & 148.62, & -47\;500, & 1000<\text{Re}<5000\\ 0.46, & -490.546, & 578\;700, & 5000<\text{Re}<10\;000\\ 0.5191, & -1662.5, & 5\;416\;700, & \text{Re}>10\;000. \end{array}\right.$$ (19)

#### 3. Droplet evaporation model

Droplet evaporation is governed by the diffusive flux of droplet vapor into the air,

$$N_{v}=k_{c}\left(C_{vd}-C_{va}\right),$$ (20)

where *N*_*v*_ is the molar evaporative flux of vapor and *k*_*c*_ is the mass transfer coefficient. *C*_*vd*_ is concentration of vapor at the saturated pressure *P*_*sat*_ on the droplet surface. The saturated pressure of water is lowered by non-volatile components such as mineral salts, and this affects the evaporation rate of the droplet. Here, we define an activity coefficient of water^34^ as the ratio of the saturated vapor pressure of pure water and water containing salt.^35^

In this case, *C*_*vd*_ is related to the saturated vapor pressure via

$$C_{vd}=\frac{P_{sat}}{RT_{d}},$$ (21)

where *T*_*d*_ is droplet surface temperature. *C*_*va*_ is then related to the partial vapor pressure,

$$C_{va}=x_{v}\frac{P}{RT_{a}},$$ (22)

where *x*_*v*_ is the species mole fraction, and *P* and *T*_*a*_ are the local pressure and temperature, respectively. The mass transfer coefficient *k*_*c*_ is correlated with the Reynolds number and the Schmidt number,^36^

$$k_{c}=\frac{D_{dv}}{D_{d}}\left(2.0+0.6\text{R}{\text{e}}^{0.5}Sc^{1/3}\right),$$ (23)

where *D*_*dv*_ is the diffusion coefficient of vapor in the air. The mass of the droplet evolves as

$$m_{d}\left(t+\Delta t\right)=m_{d}\left(t\right)-N_{v}A_{d}M_{d}\Delta t,$$ (24)

where *m*_*d*_ is the droplet mass, *M*_*d*_ is the molecular weight, and *A*_*d*_ is the surface area.

The droplet temperature is governed by thermal balance including latent and sensible heats,

$$m_{d}C_{pd}\frac{dT_{d}}{dt}=hA_{d}\left(T_{a}-T_{d}\right)-\frac{dm_{d}}{dt}h_{fg},$$ (25)

where *h*_*fg*_ is the latent heat of droplet. The convective heat transfer coefficient *h* is calculated with a modified Nusselt number,^37^

$$h=\frac{\lambda \text{ ln}\left(1+B_{T}\right)}{D_{d}B_{T}}\left(2+0.6{\text{Re}}_{d}^{0.5}\text{P}{\text{r}}^{1\;/3}\right),$$ (26)

where Pr is the Prandtl number and *λ* is the thermal conductivity of air. *B*_*T*_ is the Spalding heat transfer number,

$$B_{T}=\frac{C_{pv}\left(T_{a}-T_{d}\right)}{h_{fg}-\frac{q_{d}}{{\dot{m}}_{d}}},$$ (27)

where ${\dot{m}}_{d}$ is the droplet evaporation rate and *q*_*d*_ is the heat energy transferred to the droplet.

### B. Setup

The dimensions of the simulation domain are 10.1 (length) × 7.12 (width) × 5.4 m^3^ (height). Meshing is conducted using ANSYS FLUENT 2019 by adopting a polyhedral unstructured scheme.^38^ Steady state simulation is initially performed to obtain a converged solution for fluid flow and heat transfer as well as the concentration of species for air and vapor. Then, the steady state solution is used as an input for the transient simulation of droplet trajectories based on the discrete phase model.

**FIG. 2.**
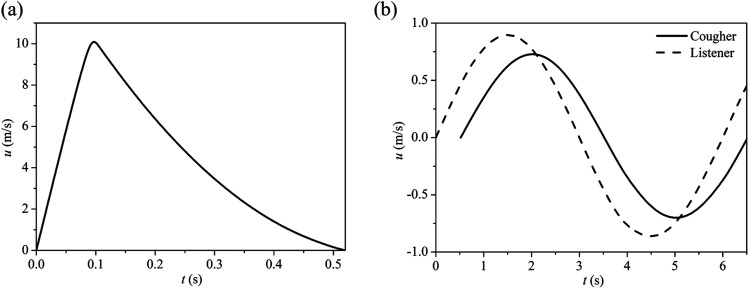
Air flow velocity for (a) cough cycle, and (b) breathing cycles for the cougher and listener. For the cougher, the breathing cycle begins at the end of the cough cycle.

In this study, we base our cough droplet size distribution on the experimental measurements reported by Duguid.^8^ The size distribution data have been verified as consistent with the other bioaerosol study elsewhere.^39^ Here, the total number of emitted cough droplets is 4897, which corresponds to a mass of 9.37 × 10^−6^ kg and a volume of 9.26 × 10^−3^ ml. Each droplet is assumed to constitute of 93.5% water and 6.5% salt in terms of mass fraction.^29^ These droplets have the same velocity as the turbulent cloud when expelled from the mouth. Based on the established cough patterns,^30^ the cough jet flow rate varies with the time. Therefore, the flow velocity also changes during a cough. The air jet velocity and the droplet velocity are obtained from the air jet flow rate by assuming a constant mouth opening area of 4 cm^2^. Figure 2 shows the velocity profiles for cough and breath cycles, respectively. The transient jet velocity profile is implemented as an user defined function (UDF) based on the ANSYS FLUENT 2019 platform.^38^ The droplets are injected into the simulation domain by using ten different injection files. Each injection file has its own droplet number and droplet velocity. The droplet number for each injection time is obtained by prorating the jet flow rates to the total jet volume based on the total expelled droplets. The droplet velocity is similar to the jet velocity at the injection time. In this work, the droplet is considered as spherical. No breakup is considered due to the low We number (maximum We number is 1.7) for the droplet size range we used.^40^ Droplet interactions are neglected due to the low number density.

## III. RESULTS AND DISCUSSION

**FIG. 3.**
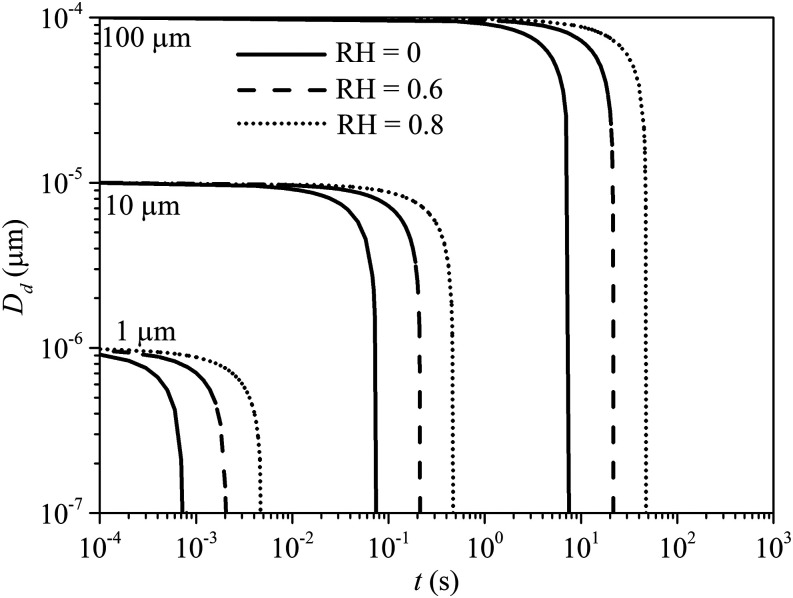
Evaporation of pure water droplets of various sizes in time under quiescent conditions. The initial droplet temperature is 37 °C, and the air temperature is 20 °C.

The results from the current numerical model were validated against the existing work for single droplet evaporation.^29,41^ Three droplet diameters, namely, 1 *µ*m, 10 *µ*m, and 100 *µ*m, are chosen to compare their evaporation time in a quiescent room under different RH. The air and droplet initial temperatures are 20 °C and 37 °C, respectively. Figure 3 shows the simulation results from the current model. In general, the results are in agreement with the works of Redrow *et al.*^29^ and Morawska.^41^

### A. Two persons 1 m apart

Two standing persons spaced 1 m apart are considered. The average climate condition during the whole day of Singapore in a year is used^42^ as a base case study: wind from behind toward the cougher at 2 m/s, an ambient air temperature 30 °C, and RH 84%. A polyhedral unstructured mesh is generated by ANSYS FLUENT 2019.^38^ The total mesh for this case is 3.9 × 10^6^. The mesh on the human surface is further refined by adding two layers of boundary mesh elements.

**FIG. 4.**
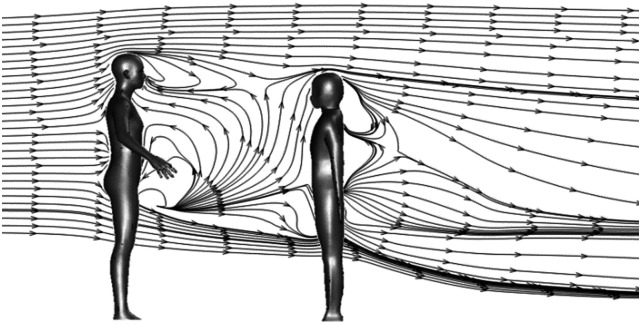
Streamline plot along the center plane of two persons spaced 1 m apart. The background wind speed is 2 m/s. The ambient air temperature is 30 °C with RH 0.84.

Figure 4 shows the streamline plots along the center plane along the flow direction. Recirculating flows, namely, wakes, are observed both in the front of the cougher and at the back of the listener. The air flow with a high velocity away from the person exerts shear stress to the air stream with a low velocity near the person, creating a wake region downstream. A droplet may be entrained and trapped in the wake, significantly altering its trajectory and fate.

In general, the motion of a droplet in air is governed by drag, inertial, and gravitational forces. Evaporation results in droplet size reduction, which modifies its flight behavior of droplets, even reversing its fate, i.e., settling to the ground or drifting with the air.

**FIG. 5.**
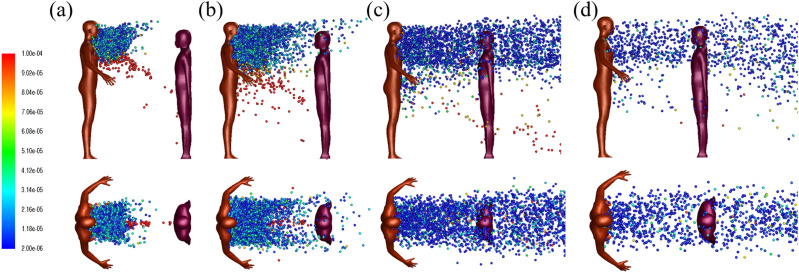
Droplet dispersion (side, top-down views) from a single cough for two persons spaced 1 m apart at (a) *t* = 0.52 s, (b) *t* = 1 s, (c) *t* = 3 s, and (d) *t* = 5 s. The jet flow angle is inclined downwards at 27.5°. The color bar indicates droplet sizes (2 *µ*m–100 *µ*m). The wind speed is 2 m/s. The ambient air temperature is 30 °C with RH = 0.84. The initial droplet temperature is 36 °C.

Figure 5 shows the snapshot of droplet trajectories at different times. The cougher stops cough at time *t* = 0.52 s and begins his normal breath cycle thereafter. The characteristic Reynolds numbers for cough is ∼13 000. Generally, large droplets separate from the turbulent cloud over time due to gravity, whereas small droplets are buoyed by the hot air cloud from the mouth and are transported over a long distance. The top-down view shows that the lateral droplet dispersion in depth is relatively constant. A slight increase for lateral dispersion is observed when the air moves around the shoulder of the listener. As the droplet diameter evolves with time during the evaporation process, the diameter here refers to the initial size of the droplet at the mouth release point. The wake entrains almost 15% of the total droplets with majorities ranging from 2 *µ*m to 75 *µ*m. Only a few droplets with diameters from 100 *µ*m to 150 *µ*m are found trapped in the wake. Most of the trapped droplets in the wakes eventually deposit on the cougher. As shown in Fig. 5(a), some of the large droplets deposit on the lower part of the listener within 0.52 s. The maximum droplet velocity is 10 m/s at *t* = 0.09 s based on the air jet velocity profiles. Despite low inhalation exposure, contamination of his/her clothes or exposed skins may lead to the secondary transmission through face, mouth, or nose touching. This result highlights potential risk for shorter persons, including children, who are less than 1 m away from a cough. This is corroborated by the findings of Dbouk and Drikakis.^20^

#### 1. Droplet size

The trajectory and fate of the droplets are affected by evaporation, which, in turn, depends on the difference between the saturated vapor pressure and the vapor pressure of the surrounding air [Eqs. (20)–(22)] and the mass diffusion coefficient [Eq. (23)]. Since the saturated vapor pressure is a function of air temperature, the droplet evaporation rate is coupled to both the temperature and humidity of the surrounding air. In general, small droplets evaporate quickly into aerosol or droplet nuclei, whereas large droplets experience longer settling times due to evaporative shrinkage.

**FIG. 6.**
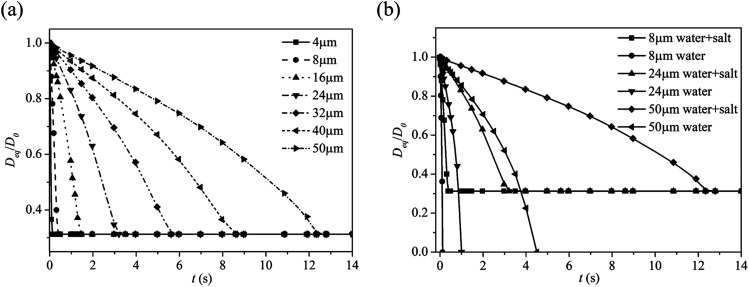
Evaporation of small droplets of (a) pure water and (b) water and salt at different times. The wind speed is 2 m/s. The ambient air temperature is 30 °C with RH = 0.84. The droplet initial temperature is 36 °C.

Figure 6 shows the evaporation time obtained by averaging droplet diameters in time grouped by their initial sizes (see the legend). Droplets with diameters 2 *µ*m or less are not included as they evaporate within a fraction of a second (∼10 ms). In particular, we found droplets ∼75 *µ*m are interesting in that different fates are observed. Roughly half of these droplets with diameters ∼75 *µ*m were suspended in the wake eventually depositing on the cougher, 45% settled to the ground rapidly due to the downward momentum of the cough jet, and the remaining 5% exited the simulation domain at around *t* = 15 s. Evaporative dynamics imbalances the competing effects of drag, inertial, and gravitational forces, which changes the fate of the droplet, i.e., settling to the ground or drifting with the air.

As shown in Fig. 6(a), the evaporation time depends significantly on different droplet diameters. For example, a 4 *µ*m droplet evaporates in 0.2 s, whereas a 50 *µ*m droplet evaporates in 12.5 s. Generally, larger droplets take a longer time to evaporate compared to smaller droplets due to its large volume to surface area ratio. Eventually, droplets evaporate into non-volatile residue or droplet nuclei, which may be involved in the airborne transmission of pathogens. These droplet nuclei are around 0.31 of the initial droplet sizes, as indicated by the horizontal lines.

The presence of salt in water reduces the water activity, which is the ratio of the partial vapor pressure of salty water and pure water, resulting in a decrease in the droplet evaporation rate. Although the exact composition of respiratory droplets is currently unclear, it is still useful to evaluate the effect of salt on the evaporation rate, since salt is one of the main components in saliva.^29^ Figure 6(b) compares evaporation times for droplets of smaller size range for both pure water and salty water. In theory, a pure water droplet evaporates completely, whereas a salty droplet evaporates into its non-volatile salt residue. Here, water activity is chosen to be 0.94 based on the mass fraction of the salt in the droplet.^34^ As shown in Fig. 6(b), the evaporation time for smaller droplets is less affected by water activity than a large one, and the evaporation time for a salty droplet is almost three times greater than that of a pure water droplet at a diameter of 50 *µ*m.

**FIG. 7.**
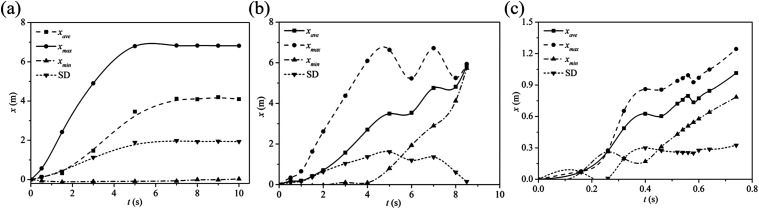
Comparison of the traveling distance for different droplet diameters: (a) 24 *µ*m, (b) 100 *µ*m, and (c) 1000 *µ*m. The wind speed is 2 m/s. The ambient air temperature is 30 °C with RH = 0.84. The droplet initial temperature is 36 °C.

Figure 7 shows the maximum, minimum, and average travel distances including standard deviations for droplets with diameters of 24 *µ*m, 100 *µ*m, and 1000 *µ*m. Most of the small droplets are carried downstream by the air flow and exit the domain, while the others remain trapped in the wake. The maximum life expectancy of a 100 *µ*m droplet is about 8.5 s with the travel distance up to 6.6 m. In comparison, the travel distance of a 1000 *µ*m droplet is 1.3 m, which, nonetheless, exceeds a social distancing of 1 m. Our findings are consistent with the more recent studies such as the work of Xie *et al.*^15^ and Bourouiba,^3^ in contrast to the older work of Wells *et al.*^13^ In addition, we note that the shrinking of the droplet due to evaporation could increase the droplet settling time and travel distance. Emphasis should be placed on mitigating the airborne transmission potential of evaporating large droplets.

#### 2. Relative humidity

Relative humidity (RH) is the ratio of the partial pressure of vapor to the saturated vapor pressure of water at a given temperature. At low RH, air has a low partial vapor pressure, resulting in a large pressure difference on the droplet surface and fast evaporation rates. The evaporation rates depend on competing inertia, gravitational, and drag forces and play an important role in not only the droplet trajectory but also the viability of its viral content.

**FIG. 8.**
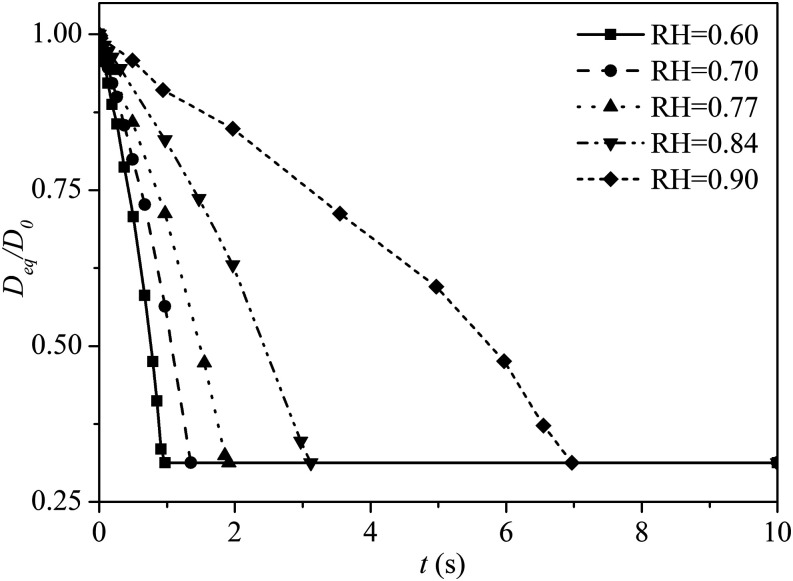
Average evaporation rate for the droplet with 24 *µ*m under different RH. The wind speed is 2 m/s. The ambient air temperature is 30 °C. The droplet initial temperature is 36 °C.

Typically, the RH in a tropical climate, such as in Singapore, fluctuates between 0.60 and 0.94 during different times of the year. Here, five different values of RH are selected for comparison, namely, 0.60, 0.70, 0.77, 0.84, and 0.90. For simplicity, we have fixed the temperature at 30 °C. Figure 8 shows the average evaporation rate of the 24 *µ*m droplet under different RH. The evaporation time, before a droplet dries out, is increased significantly under high RH conditions. By inspection, the evaporation time for a droplet at RH = 0.90 is approximately seven times higher than that of the one at RH = 0.60. Under low humidity conditions, a small droplet evaporates rapidly into smaller residual nuclei, which can remain airborne for hours. Therefore, pathogens within these droplet nuclei may present a greater long range transmission threat than the droplets.

**FIG. 9.**
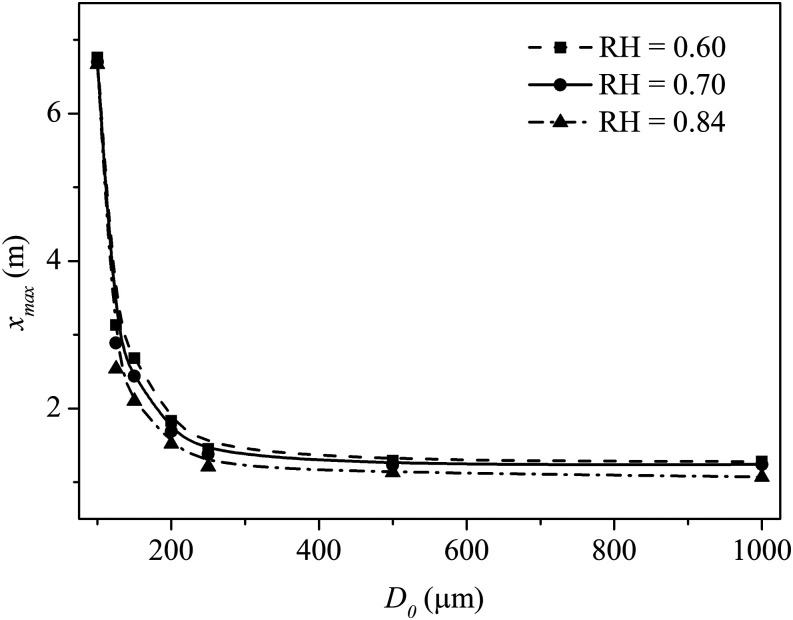
Effect of RH on the maximum lifetime for different large droplets. The wind speed is 2 m/s, the ambient air temperature is 30 °C, and the droplet initial temperature is 36 °C.

Figure 9 shows the effect of RH on the maximum travel distance for large droplets ranging from 100 *µ*m to 1000 *µ*m. Large droplets evaporate slower than the smaller ones due to their greater volume to surface area ratios. In addition, gravitational forces are predominant for large droplets, and so they tend to settle quickly. Between droplets of similar sizes, the travel distance at lower RH is slightly greater than at higher RH. At low RH, a droplet has a high evaporation rate and shrinks quickly, leading to a longer life expectancy and travel distance. As shown in Fig. 9, the travel distance of a 100 *µ*m droplet is almost double that of 125 *µ*m. Significantly, a 1000 *µ*m droplet can be found more than 1 m away, regardless of RH.

**FIG. 10.**
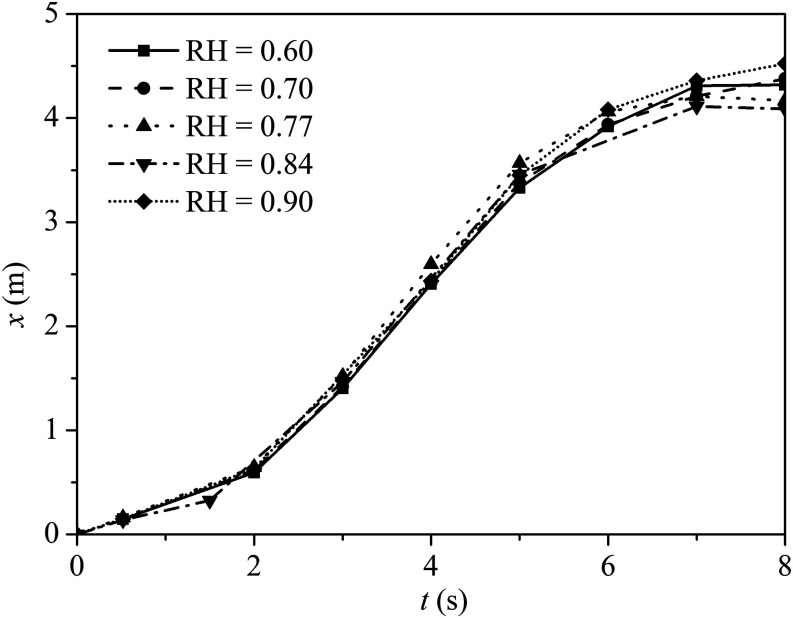
Average travel distance for the droplet with a diameter of 24 *µ*m under different RH from 0.60 to 0.94. The wind speed is 2 m/s, the ambient air temperature is 30 °C, and the droplet initial temperature is 36 °C.

The average travel distance for a 24 *µ*m droplet under different RH is shown in Fig. 10. We note that the dispersion distances are relatively insensitive to RH. In addition, these droplets evaporate into droplet nuclei and may drift in the air for hours. Since there is no clear evidence for the dilution and inactivation of airborne SARS-CoV-2 viruses over a long period of time, there is significant potential for airborne transmission under low humidity conditions.

#### 3. Wind speed

The effect of wind speed in the outdoor environment is discussed. The case in point, the average monthly surface wind speed in Singapore is from 1.5 m/s to 3.1 m/s in a year.^38^ Three different wind speeds including 1 m/s, 2 m/s, and 3 m/s are studied in the current work. The winds are from behind the cougher with an ambient air temperature and a relative humidity of 30 °C and 84%, respectively. In each simulation, the cougher resumes a normal breathing cycle at the end of the cough, while the Listener breathes normally for the entire duration of the study. An additional simulation is performed for the wind speed of 0 m/s as the control case of windless condition.

**FIG. 11.**
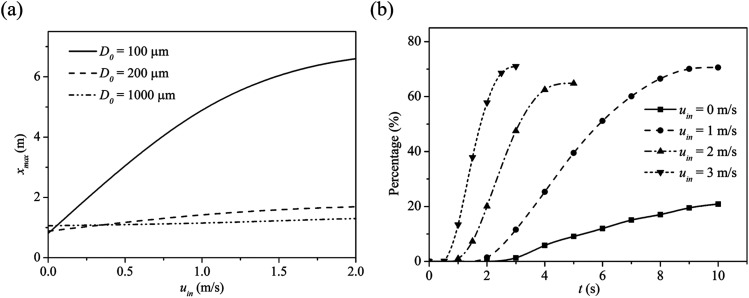
(a) Effect of wind speed on the maximum travel distance for droplets of indicated sizes. (b) Fraction of droplets with the horizontal distances exceeding 1 m distance from the source at 10 s. The ambient air temperature is 30 °C with RH = 0.84, and the droplet initial temperature is 36 °C.

At greater wind speeds, small airborne droplets are projected to greater distances, and so potential transmission risks increase with the wind speed. Figure 11(a) shows the maximum travel distance for three selected droplet sizes, i.e., 100 *µ*m, 200 *µ*m, and 1000 *µ*m, under different wind speeds. As the droplets are expelled by a forceful cough, the trajectories of large droplets are mainly driven by the inertial and gravitational forces. Under still air conditions, i.e., *u*_*in*_ = 0 m/s, larger droplets can travel further than the smaller ones, and the 1000 *µ*m droplet has the greatest travel distance among the droplets. Under windy conditions, the drag forces applied by the moving air on the droplets may be significant. Generally, inertial, gravitational, and drag forces, coupled with evaporative physics, determine the airborne dynamics and the travel distance of these droplets. For wind speeds *u*_*in*_ > 0.1 m/s, the 100 *µ*m droplet is found with the longest travel distance among all the droplets. This is attributed to two reasons: first, a 100 *µ*m droplet evaporates and shrinks faster than the other droplets, and so they become smaller and lighter. Second, the life expectancy of a 100 *µ*m droplet is much longer than the others, and so it can remain airborne and thus disperse further.

Figure 11(b) shows the fraction of droplets that are at least 1 m from the source at 10 s from the onset of the cough. Under windless conditions, i.e., *u*_*in*_ = 0 m/s, the breathing cycles could still generate sufficient air motion to disperse small droplets, although no droplet is found further than 2 m within the simulation time. At the wind speed *u*_*in*_ = 1 m/s, some droplets exit the simulation domain at *t* = 10 s. With a high air velocity, the droplets exit from the simulation domain in a short time. Despite the lowest number of droplets exceeds 1 m at stationary flow, the number is almost 20% of the total droplet expelled by the cougher among which most of them are large droplets. Interestingly, the fraction of droplets exceeding 1 m is smaller at *u*_*in*_ = 2 m/s than either *u*_*in*_ = 1 m/s or 3 m/s. This may be due to the droplets trapped in the wake before the cougher, which is generated at a substantial wind speed *u*_*in*_ = 2 m/s, but at even faster wind speeds *u*_*in*_ = 3 m/s, the droplets could carry sufficient momentum to escape from the wake.

### B. Two persons 2 m apart

**FIG. 12.**
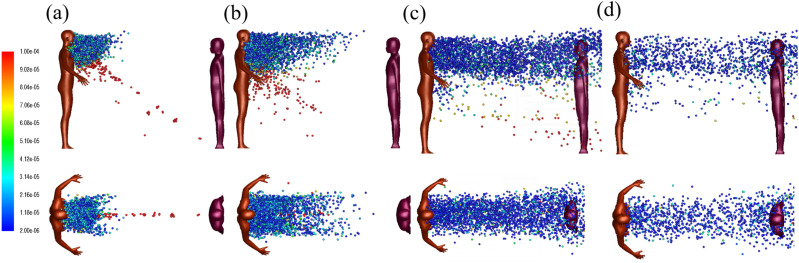
Droplet dispersion (side and top-down views) for two persons spaced 2 m apart at (a) *t* = 0.52 s, (b) *t* = 1 s, (c) *t* = 3 s, and (d) *t* = 5 s. The wind speed is 2 m/s. The ambient air temperature is 30 °C with RH = 0.84. The droplet initial temperature is 36 °C.

In this scenario, the two persons are now spaced 2 m apart instead of 1 m under otherwise identical conditions. Figure 12 shows the instantaneous snapshots of droplet dispersion at various times. Large droplets travel faster and further than small droplets, and they are separated from the droplet cloud; some of these large droplets are already near the listener by 0.52 s. As expected, the droplets take a longer time to reach the listener who is now 2 m away instead of 1 m, but it also seems that the droplet dispersion is not affected by presence of the listener at this distance. Finally, we observe that the increased dispersion width at 2 m significantly reduces the number density of droplets that reach the listener, with implications on deposition as explained in Sec. III B 1.

#### 1. Infection risk assessment

We have collected the droplet deposited on the listener during simulation. To assess the transmission risk, the median SARS-CoV-2 viral load (3.3 × 10^6^ copies/ml) of saliva specimens tested by To *et al.*^43^ is used here to analyze the infection risk of the listener. Note that the viral load obtained by To *et al.*^43^ is based on saliva without considering water evaporation. Therefore, the droplets’ sizes deposited on the Listener from the simulation results are based on their initial size. The viral load concentration is assumed to be uniform among all droplets, and so the viral load in a droplet is proportional to its volume. Such an assumption is consistent with the work of Smith *et al.*^44^ where they concluded that large droplets may contain more virus particles per droplet compared with aerosol droplets.

**FIG. 13.**
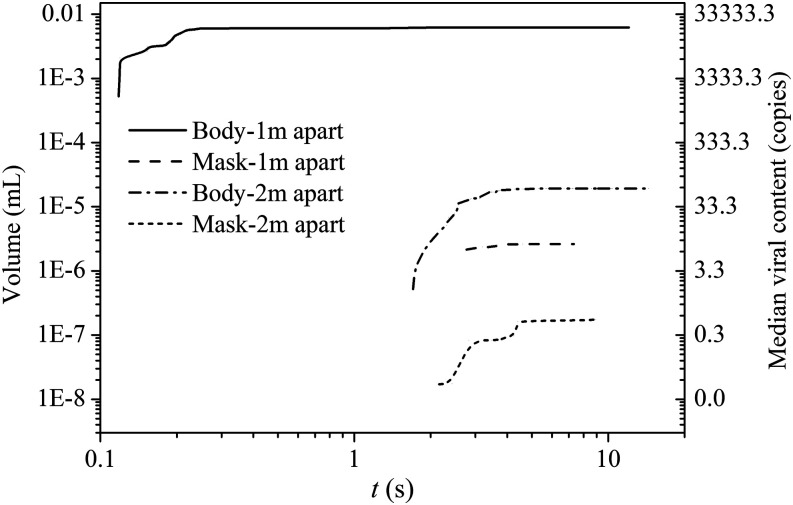
Droplets and virus load collected on the listener model surface, including body (1.43 m^2^) and mask (288 cm^2^).

The left and right *y*-axes in Fig. 13 show the accumulative total volume of the droplets and the viral load landed on the listener body and mask with the social distancing of 1 m and 2 m, respectively. More than 65% of the droplet volume expelled by the cougher is deposited on the listener when the two persons are spaced 1 m apart with a tremendous viral load. The droplet size covered almost all the range of expelled droplets. In fact, 68% of the droplets deposited on the listener are large droplets with a diameter larger than 100 *µ*m. The volume fraction for these large droplets is around 99% of the total volume deposited on the listener. These droplets are mainly distributed on the lower part of the listener. They are unlikely inhaled by the listener through the respiratory system. However, the contamination of listener’s clothes, especially the exposed skins due to these droplets, should not be overlooked. This may lead to the secondary transmission via face, mouth, or nose touching. With the social distancing elevated to 2 m, the droplet volume landed on the listener’s body reduced significantly. The total volume is about 2.0 × 10^−5^ ml with the viral load around 63 copies. The droplet size deposited on the listener ranged from 2 *µ*m to 150 *µ*m among which 90% of the droplet volume is caused by the large droplet. Studies of the SARS-CoV-2 virus reveal that no culture was obtained from the samples with the critical cycle threshold (Ct) value larger than 34.^45,46^ The corresponding viral load to this critical Ct value is around 60 copies or more for risk of infection. Jones *et al.*^47^ concluded that physical distancing is not sufficient for mitigating the transmission of SARS-CoV-2 and should be supplemented with other hygiene measures such as self-hygiene and cleaning. As shown in Fig. 13 in our study, we also found that even the two persons are spaced 2 m apart, the viral transmission cannot be underestimated. As most of the droplets are large droplets that could probably lead to the secondary transmission risk, this can be minimized by taking proper self-hygiene measures such as washing of hands, exposed surfaces, and clothes after outdoor activities.^48^

Respiratory infections such as SARS-CoV-2 can be transmitted through droplets or aerosols with a diameter smaller than 10 *µ*m. Small aerosols (less than 5 *µ*m) penetrate deep into the lung, while large droplets (5 *µ*m–10 *µ*m) are generally trapped in the upper respiratory system. For mask-wearing individuals, we designated an area on the listener’s mouth and nose as a mask to account for droplet deposition on it. The area of the mask is 288 cm^2^ with the aspect ratio of 1.43. The results show that fewer droplets deposit on a mask compared to the body, and they are generally droplets with small diameters. The viral loads on the mask reduced from 9 copies to 0.6 copies when the social distancing is from 1 m to 2 m, respectively. Although the viral loads are reduced significantly for outside-in transmission, mask wearing is important for inside-out transmission. In addition, the potential infection risk cannot be overlooked in cases where the infected cougher has series of coughs or at a higher viral load compared to the median levels.

#### 2. Limitations

The present study has several limitations, including the following:

(1) The cough model is idealized where droplet velocities for both large and small droplets are assumed to be similar at the source. Further study is necessary to determine the effects of initial droplet velocity for each size group on dispersion.
(2) Currently, our droplet size distribution, based on the experimental measurements from Duguid,^8^ only covers one size interval between 50 *µ*m and 100 *µ*m. A more refined study is required to evaluate the effects of evaporation on droplet sizes between 50 *µ*m and 100 *µ*m.
(3) The effects of ambient temperature and humidity on the viability of SARS-CoV-2 are unclear. Further studies should be made on this aspect.
(4) The risk assessment entailed in this study pertains only to idealized environmental conditions, in particular, a specific reference viral load.^43^ These limiting factors may evolve as the SARS-CoV-2 situation develops.

## IV. SUMMARY

We modeled fluid flow and droplet dispersion from a respiratory event, in this case, a cough, in a tropical outdoor environment. The effects of relative humidity, wind speed, and social distancing on the droplet dispersion are investigated. Further analysis of the droplet volume deposited on the listener as well as the viral load in terms of SARS-CoV-2 carried by these droplets are discussed. Comparisons are made between the social distancing of 1 m and 2 m to assess the infection risk.

Highlights and recommendations are as follows:

1. Droplets less than 50 *µ*m in diameter can remain airborne over long distances. Droplets larger than 75 *µ*m settle in trajectories following the downward jet profiles shown in Fig. 2(a). At wind speeds of 2 m/s, travel distances for droplet sizes 100 *µ*m and 1000 *µ*m are 6.6 m and 1.3 m, respectively, at 30 °C and RH 0.84. The travel distances for large droplets cannot be underestimated.
2. The presence of non-volatile components generally reduces the evaporation rate of a droplet. The evaporation time of a 50 *µ*m droplet is 4.5 s for pure water and 12.5 s for salt mass fraction of 6.5% at 30 °C and RH 0.84. For small droplets, we show that increasing RH from 0.60 to 0.90 does not result in significant difference in dispersion distances at the same temperature. For large droplets, shrinkage due to evaporation results in an increase in lifetime and travel distance. Stronger effects of evaporation on dispersion are observed for droplets smaller than 300 *µ*m.
3. Large droplets may travel more than 1 m under windless conditions. The travel distance correlates well with the wind speed. For a 100 *µ*m droplet, the travel distance increases from 0.8 m without wind to 6.8 m at a wind speed of 3 m/s.
4. Social distancing is generally effective at reducing the droplet volume as well as the viral load deposited on the listener. Although the inhaled viral load of 9 copies to 0.6 copies seems small under the social distancing of 1 m and 2 m, these numbers are only based on the median viral load. Viral exposure could increase significantly through successive coughs or higher viral loads.
5. Droplet deposition on skin and clothes may not directly lead to infection. However, secondary transmission modes, including face, mouth, or nose touching, need to be avoided. Hygiene measures such as washing of hands and exposed surfaces are highly recommended.
6. Young children may be at greater risk compared to adults based on the typical downward cough trajectory. Teenagers and short adults are advised to maintain a social distance greater than 2 m from taller persons. Surgical masks are known to be effective at trapping large droplets and therefore recommended for use as necessary.

## Data Availability

The data that support the findings of this study are available from the corresponding author upon reasonable request.
